# Short-term efficacy and safety of low-intensity extracorporeal shock wave therapy in erectile dysfunction: a systematic review and meta-analysis

**DOI:** 10.1590/S1677-5538.IBJU.2016.0245

**Published:** 2017

**Authors:** Zi-jun Zou, Liang-you Tang, Zhi-hong Liu, Jia-yu Liang, Ruo-chen Zhang, Yu-jie Wang, Yong-quan Tang, Rui Gao, Yi-ping Lu

**Affiliations:** 1 Department of Urology, Institute of Urology, West China Hospital, Sichuan University, Chengdu, Sichuan, P.R. China; 2 Department of Urology, the First Affiliated Hospital of Fujian Medical University, Fuzhou, Fujian, P.R. China

**Keywords:** Erectile Dysfunction, Therapeutics, Meta-Analysis as Topic

## Abstract

**Aim:**

The role of low-intensity extracorporeal shock wave therapy (LI-ESWT) in erectile dysfunction (ED) is not clearly determined. The purpose of this study is to investigate the short-term efficacy and safety of LI-ESWT for ED patients.

**Materials and Methods:**

Relevant studies were searched in Medline, Embase, Cochrane Library, China National Knowledge Infrastructure (CNKI), WANFANG and VIP databases. Effective rate in terms of International Index of Erectile Function-Erectile Function Domain (IIEF-EF) and Erectile Hardness Score (EHS) at about 1XSmonth after LI-ESWT was extracted from eligible studies for meta-analysis to calculate risk ratio (RR) of effective treatment in ED patients treated by LI-ESWT compared to those receiving sham-treatment.

**Results:**

Overall fifteen studies were included in the review, of which four randomized controlled trials (RCTs) were for meta-analysis. Effective treatment was 8.31 [95°/o confidence interval (CI): 3.88-17.78] times more effective in the LI-ESWT group (n=176) than in the sham-treatment group (n= 101) at about 1 month after the intervention in terms of EHS, while it was 2.50 (95% CI: 0.74–8.45) times more in the treatment group (n= 121) than in the control group (n=89) in terms of IIEF-EF. Nine-week protocol with energy density of 0.09mJ/mm^2^ and 1500 pluses seemed to have better therapeutic effect than five-week protocol. No significant adverse event was reported.

**Conclusion:**

LI-ESWT, as a noninvasive treatment, has potential short-term therapeutic effect on patients with organic ED irrespective of sensitivity to PDE5is. Owing to the limited number and quality of the studies, more large-scale, well-designed and longterm follow-up time studies are needed to confirm our analysis.

## INTRODUCTION

Erectile dysfunction (ED) is a common male sexual dysfunction and oral phosphodiesterase type 5 inhibitor (PDE5i) is a first-line therapy (1). Although ameliorating erectile function (EF) significantly, PDE5is are not curative approaches and patients have to plan sexual activity with the aid of medication. In addition, a part of ED patients poorly respond to PDE5is and need to turn to invasive treatments such as intra-cavernosal injection of vasoactive agents and surgical implantation of penile prostheses. Therefore, a novel treatment that improves EF in a noninvasive and enduring manner is required.

Shockwave is characterized by acoustic wave generating pressure impulses. It has been used widely in the field of medicine, where the role of shockwave therapy varies with the level of energy intensity (2). Different from high- and medium-intensity shockwave with focused mechanical destructive and anti-inflammatory nature, low-intensity extracorporeal shock wave therapy (LI-ESWT) probably has angiogenic property based on resultant cell membrane microtrauma and mechanical stress that are associated with the release of angiogenic factors (3) and recruitment of circulating endothelial progenitor cells (4). Therefore, LI-ESWT has been used for vasculogenic disease containing peripheral artery disease (5), chronic wounds (6), and cardiac ischemic diseases (7).

Based on the potential stimulation of angiogenesis and local vascularization (8), LI-ESWT was also used for vasculogenic ED and had considerable effectiveness in terms of sexual performance, penile blood flow and endothelial function (9-11). It will hopefully make up the defects in the treatment of ED given that 1) the potential property of altering spontaneous erectile function in an enduring and pathophysiological way (9); 2) reversing insensitivity to PDE5is (11). Meanwhile, penile LI-ESWT was proved to be safe during and after the treatment in the pilot studies (9-11).

Although underlying mechanism is still under investigation, LI-ESWT has been listed in the chapter of first-line therapy since 2013 European Association of Urology (EAU) guidelines on male sexual dysfunction (1), supported by a series of prospective trials containing randomized controlled trials (RCTs) (9-11). Nevertheless, defined recommendation cannot be given because current evidences are relatively limited. Recently, two systematic reviews and meta-analyses (12, 13) on the topic were published. However, the result of the study by Lu et al. (12) was less convincing because significant heterogeneity existed among included studies with ED patients originating from different pathology, and the evidence level of Angulo's study (13) was lowered by including single-arm trials. Therefore, a systematic review and meta-analysis focusing on RCTs regarding LI-ESWT for organic ED without Peyronie's disease (PD) and chronic pelvic pain (CPP) is essential.

## MATERIALS AND METHODS

The systematic review and meta-analysis was performed following PRISMA criteria (14).

### 

#### Criteria for study inclusion/exclusion

Studies, which contained RCT, single-arm trial and respective study, reporting LI-ESWT in the management of ED patients without PD and CPP, were included for this systematic review. If data regarding effective and/or complication rate could be extracted, those included RCTs were further performed for meta-analysis. In addition to eligible original articles, reviews in the field were also identified for further searching of reference lists to ensure the completeness of the literature search. Case reports, letters to the editor, conference abstract, comment and basic studies were excluded.

Two authors reviewed the included articles independently. Disagreements were resolved by discussion and consensus. Duplicate publications were excluded, and when different literatures discussed a same cohort, the most informative one was used for further analysis.

#### Search strategy

Two authors independently searched Medline, Embase, Cochrane library and Chinese medical electronic databases including China National Knowledge Infrastructure (CNKI), WANFANG and VIP by using one of “shockwave” and “shock wave” combined with one of “erectile dysfunction” and “ED” as a search term with overall 4 combinations. English and Chinese literatures between January 2010 and December 2015 were included. The reference lists of eligible studies and relevant reviews were searched in case of possible missing articles.

#### Assessment of risk of bias in included studies

Risk of bias in the RCT was assessed according to the Cochrane Collaboration's tool for assessing risk of bias (15), which addresses sequence generation, allocation concealment, blinding, handling of incomplete data, and selective reporting. The quality of other studies was assessed by the Methodological Index for Non-randomized Studies (MINORS) (16) and global ideal score is 16 points for non-comparative study.

#### Data extraction

Data extraction was done by two authors independently, and disagreements were resolved by discussion. Titles and abstracts were used to screen for initial study inclusion. Full-text review was carried out on the remaining papers that matched inclusion/exclusion criteria. The same reviewers performed all data extraction including study characteristics and outcome data. A data-extraction form was used with variables containing author, publication year, type of study, country, LI-ESWT protocol, ED type, previous sensitivity of PDE5i, endpoints, and outcomes.

In the meta-analysis, effect size (ES) was risk ratio (RR) of effective treatment in ED patients receiving LI-ESWT and sham treatment. International Index of Erectile Function-Erectile Function Domain (IIEF-EF) score and Erectile Hardness Score (EHS) were validated and most widely used in clinical trials to evaluate erectile function. The numbers of total participants and effective ones measured with them were extracted from included literatures. Short-term effective treatment was defined as 5-point or greater improvement in the IIEF-EF between baseline and score at about 1 month after LI-ESWT or an increase in EHS from 2 or less at baseline to 3 or more at about 1 month after the intervention. The definitions were accepted because most participants and studies could be included. Adverse event outcome was also summarized and analyzed.

### Statistical analysis

Random effects model was selected for the calculation of RR based on acknowledging heterogeneities in our samples with several protocols of LI-ESWT and populations with different sensitivity of PDE5is. A chi-squared test was conducted for heterogeneity evaluation and p value less than 0.05 was considered to be statistically significant. I^2^ index was used to quantify between-study heterogeneity's contribution to overall heterogeneity. Funnel plot was conducted for evaluation of publication bias. Sensitivity analysis was conducted to explore the heterogeneity via excluding every study one by one. Subgroup analysis according to different protocols, energy densities and doses of LI-ESWT, the consistency of risk factors between the treatment and control group, and sensitivity to PDE5is, was conducted. Statistical calculation was conducted using Review Manager (version 5.3) software.

## RESULTS

### 

#### Literature search results

**Figure 1:**
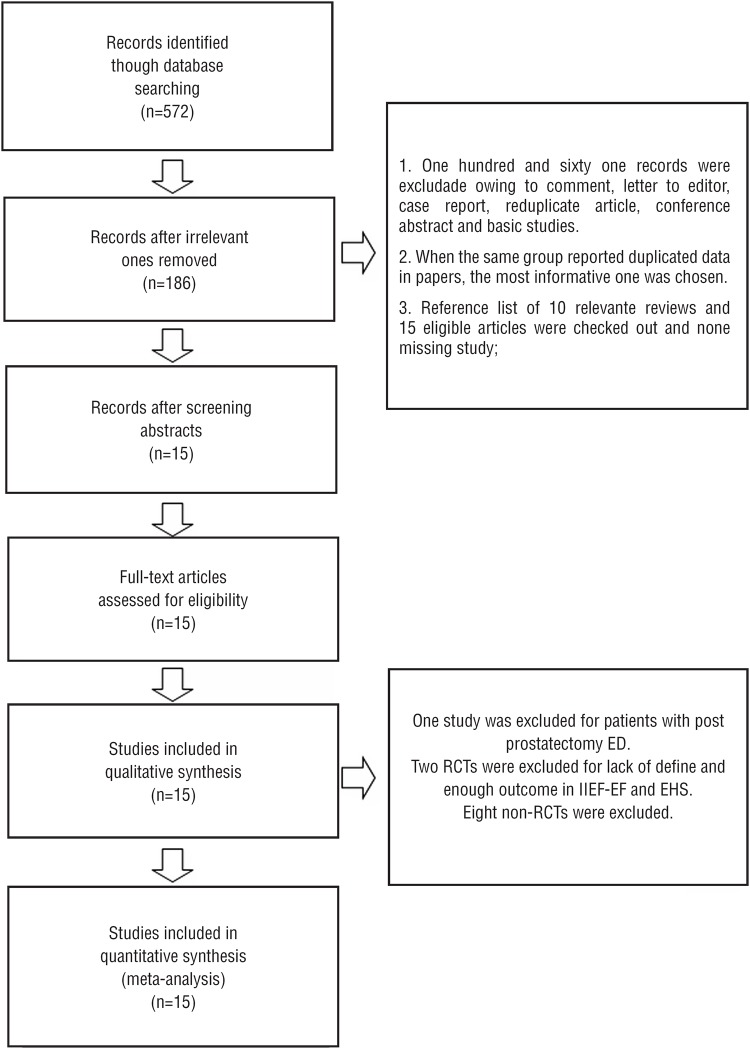
Flow diagram outlining search results and final included and excluded studies.

The search process is shown in Figure-1. The first search yielded 572 potentially relevant studies, of which 386 were irrelevant and were excluded after reviewing their titles. Abstracts of the remaining 186 studies were considered for detailed evaluation. One hundred and sixty one studies were excluded in that stage due to reduplicate cohorts, and being letter to the editor, case report, conference abstract and comment. No more eligible articles were found in search of the reference lists of 10 relevant reviews and the 15 original literatures.

Finally, 15 original articles (9-11, 17-28) were included in the systematic review after full-text evaluation. Table-1 shows the characteristics of the eligible studies with 6 RCTs (10, 17, 18, 21, 25, 28) and 9 prospective, single-arm trials (9, 11, 19, 20, 22-24, 26, 27). There was a study (27) about ED secondary to nerve-sparing prostatectomy, while the other studies focused on organic ED that was mainly considered to be vasculogenic. Although the protocol, device, energy density and dose of LI-ESWT were not uniform, all treatments had positive effects on ED in the studies. The details of the methods were summarized in Table-2. A RCT from China (21) compared the efficacy of LI-ESWT and vacuum erectile device for ED. With two RCTs (18, 21) with lack of sufficient data, overall 4 RCTs (10, 17, 25, 28) were included for quantitative synthesis.

**Table 1:** Characteristics of the included studies in the systematic review.

ID	Study	Country	Sensitivity to PDE5i	No. of patients	Intervention+	End points	Outcomes
Vardi Y. 2010 (9)	Single arm	Israel	Responders	20	1; without PDE5i	A change in the IIEF-ED domain score of >5 points was used as the main measure of treatment success.	At 1 mo follow-up, 1) 20.9±5.8 vs. 13.5±4.1(baseline), p < 0.001 in IIEF-ED scores remaining unchanged at 6 mo; 2) Significant increasing in the duration of erection and penile rigidity, and significant improvement in penile endothelial function; 3) Ten men did not require any PDE5-I therapy after 6-mo follow-up.
Vardi Y. 2012 (10)	RCT	Israel	Responders	40 (treatment) vs. 20 (placebo)	1; without PDE5i	**Primary end point:** A 5-point or greater improvement in the IIEF-EF between baseline and at 4 w after treatment. **Secondary end point:** Significant increase in the IIEF subcategories. An increase in EHS from ≤2 at baseline to ≥3 at 4w after treatment, and an improvement in penile blood flow.	1) Increase in IIEF-EF score: 6.7±0.9 (LI-ESWT) vs. 3.0±1.4 (sham), p=0.0322; 2) 19 (LI- ESWT) vs. none (sham) in patients with baseline EHS ≤2 having EHS≥3 after treatment; 3) 8.2 vs. 0.1 ml/m/dl in FMD, p< 0.0001.
Gruenwald I. 2012 (11)	Single arm	Israel	Non-responders	29	1; without PDE5i at 4w after completing LI-ESWT (FU1) and use it after 8w (FU2).	Change in IIEF-ED, EHS and three parameters of penile hemodynamics and endothelial function.	1) Mean IIEF-ED scores increased from 8.8±1 (baseline) to 12.3±1 at FU1 (P = 0.035). At FU2 (on active PDE5i treatment), their IIEF-ED further increased to 18.8±1 (P < 0.0001); 2) 72.4% (P < 0.0001) reached an EHS of≥3; 3) A significant improvement (P = 0.0001) in penile hemodynamics and this improvement significantly was correlating with increases in the IIEF-ED (P < 0.05).
Olsen A.B. 2014 (17)	RCT	Denmark	Responders	51 (treatment) vs. 54 (placebo)	5; without PDE5i	**Primary end point:** The treatment success threshold was set at EHS 3-4. **Secondary end point:** An increase in IIEF-EF domain score of at least 5 points.	Twenty-nine men (57%, active group) were able to have sexual intercourse without the use of medication vs. 5 men (9%, placebo group, p = 0.0001) after 5 weeks of completing LI-ESWT. But no significant result was found with the use of the IIEF-EF.
Yee C.H. 2014 (18)	RCT	China	Unknown	30 (treatment) vs. 28 (placebo)	1; whether other modality being used was unknown.	**Primary end point:** The 13-week change from baseline for IIEF-ED score after one course of Li-ESWT. **Secondary end point:** The interval change of EHS and adverse events from LI-ESWT therapy.	At 4w follow-up, 1) mean IIEF-ED score: 17.8±4.8 (LI-ESWT) vs. 15.8±6.1 (sham), p=0.156; 2) mean EHS: 2.7±0.5 (LI-ESWT) and 2.4±0.9 (sham), p = 0.163.
Bechara A. 2015 (19)	Single arm	Argentina	Non-responders	25	3; use PDE5i	Whenever patients improved on all IIEF-6, SEP2 and SEP3 and to respond positively to the GAQ at 3 months post-treatment.	60% (12/20) of the patients responded to the treatment.
Chung E. 2015 (20)	Single arm	Australia	Failed or unsatisfactory outcome with oral PDE5i and/or vasoactive agents	30	4; Whether other modality being used was unknown.	Change in IIEF-5 and EDITS scores, and overall satisfaction rate were recorded at 6 weeks and 4 months after completion of LI-ESWT.	At 6 weeks and 4m, 60% of patients reported an improvement in IIEF-5 score by 5 points, 70% improvement in EDITS Index score by > 50%. 67% of patients satisfied (scoring 4 out of 5) and 80% would recommend the therapy.
Qi T. 2015 (21)	RCT	China	Unknown	30 (LI- ESWT) vs. 30 (vacuum erectile device)	7; unknown	At 1 mo after LI-ESWT. 1) Cure: IIEF-5 score ≥ 22pts, or SEP, GAQ and EHS is 5, 2 and 4pts, respectively; 2) Relief: when IIEF-5 score<22pts, a 5 point or greater improvement in the IIEF-5, or SEP≥4pts, GAQ≥1pts, EHS≥3pts; 3) Fail: IIEF-5 score<21pts and improvement score ≤4pts, SEP<3pts, GAQ=0pts, EHS<2pts.	The number of cured patient was 14 and the number of relief was 8. Effective rate was 73% (22/30) in LI-ESWT group.
Pelayo-Nieto M. 2015 (22)	Single arm	Mexico	Unknown	15	3; unknown medication history	In IIEF-EF, success of treatment was defined as an increase of >2 points and >5 points in groups of mild and moderate, respectively. Results were evaluated by using IIEF, EHS, SEP, GAQ at 1 and 6 months after treatment.	The rate of success was 80%. 1) IIEF: 15 (11-18) pts at baseline vs. 20 (11-23) pts at 1 and 6 mo, p<0.013; 2) EHS: 2 (2-3) pts at baseline vs. 4 (2-4) pts at 1 mo, p<0.01; 3) SEP3:7 patients at baseline vs. 12 patients at 1 mo, p=0.0013.
Reisman Y. 2015 (23)	Single arm	Netherlands, et al	Responders and Non-responders	58	2; without PDE5i until 1 month post treatments.	**Primary end point:** An increase of IIEF-EF score from baseline to the third follow-up (6m post treatment) according to the initial ED severity: >2-point increase for mild symptoms; >5 points for moderate symptoms; and >7 points for severe symptoms.	47(81%) had a successful treatment.
Ruffo A. 2015 (24)	Single arm	Italy	Non-responders	31	2; without PDE5i during treatment.	**Primary end point:** An increase of IIEF-EF score from baseline to 1 and 3 months after LI-ESWT. **Secondary end point:** Improvement in SEP2, 3 and GAQ.	1) IIEF-EF: 16.54±6.35 (baseline) vs. 21.13±6.31 (1 mo), 21.03±6.38 (3 mo). 2) SEP2 (yes): 61% (baseline) vs. 86% (1mo). 89% (3 mo). 3) SEP3 (yes): 32% (baseline) vs. 58% (1mo), 62% (3 mo); all p<0.05.4) GAQ: at 1 and 3 mo, difference is not significant.
Srini V.S. 2015 (25)	RCT	India	Responders	60 (treatment) vs. 17 (placebo)	1; without PDE5i	**Primary end point:** ≥5 points improvement in the IIEF-EF between baseline and 1 mo (also 12 mo). **Secondary end point:** Significant increase in the CGIC and an increase in EHS from ≤2 at baseline to ≥ 3 at FU1 and FU5.	1) Increase in IIEF-EF: at 1 mo, 12.5 pts in LI- ESWT group vs. 1.4 pts in control group; at 12 mo. 8.7 pts in LI-ESWT group vs. NA in control group. 2) Effective rate in EHS: 90% (1m), 83% (12m) vs. none (placebo group). 3) Data about CGIC were not provided.
Hisasue S. 2016 (26)	Single arm	Japan	Unknown	56	1; use PDE5i on-demand after LI-ESWT.	Assessing the patients with SHIM, EHS, and MPCC at 1, 3 and 6 months after the final LI-SWT.	64.2% patients showed improvement in SHIM scores, and 57.1% patients achieved an EHS 3 or 4 without PDE5i within 6 months after LI-SWT. MPCC showed significant improvement in 64% patients from 1 month after treatment, maintaining it until 6 months.
Frey A. 2016 (27)	Single arm	Denmark	Postprostatectomy ED with unknown sensitivity to PDE5i	16	6; use of erectogenic aids	**Primary end point:** Changes in IIEF-5 scores. **Secondary end point:** A global satisfaction question ranging from “very dissatisfied” to “very satisfied”.	The median change in IIEF-5 scores was +3.5 (range −1 to 8; p=0.0049) and +1 (range −3 to 14; p=0.046); 11 and 7 patients reported being either satisfied or very satisfied at 1 mo follow up and 1 year follow up, respectively.
Kitrey N.D. 2016 (28)	RCT	Israel	Non-responders	37 (treatment) vs. 18 (placebo)	1; use PDE5i when evaluating results.	**Main outcomes:** 1) EHS was 3 or greater; 2) A change in IIEF-EF was greater than 7 points for severe ED and 5 points for moderate ED. **Secondary outcome:** FMD penile time-flow AUC as an indicator of penile endothelial function and the CGIC questionnaire. They were evaluated at 1 month after the end of treatment.	1) 54.1% (LIST) vs. none (sham) had EHS=3, p<0.0001; 2) in IIEF-EF, 40.5% (LIST) vs. none (sham), p=0.001; 3) 56.3% of the patients treated with active LIST after sham treatment achieved an erection hard enough for penetration (p<0.005); 4) The change in penile hemodynamic parameters was statistically significant; 5) According to CGIC, 56.8% of patients (LIST) vs. 27.8% (sham)(p=0.051) reported clinical improvement.

+ Number in the column of intervention represents different protocol of LI-ESWT and is consistent with the ID in table 2.

**Table 2:** The reported protocols of LI-ESWT in included studies.

ID	Device	Energy density	Frequency	Distribution of energy	Cycle of treatment
1	Omnispec ED1000 (Medispec Ltd., Yehud, Israel / Germantown, MD, USA)	1500 shocks of 0.09mJ/mm^2^	120 shocks /min	300 shocks were delivered at each of the 5 treatment points (the distal, mid and proximal penile shaft, and to the left and right crura).	Nine-week treatment period: two LI-ESWT sessions per week for 3 weeks, repeated after a 3-week no treatment interval / twice a week for 4 weeks
2	Renova ®(Direx Group LTD)	3600 shocks of 0.09mJ/mm^2^	a maximum rate of 300 shocks/ min	900 shocks were delivered at each of the 4 treatment points (left and right corpus cavernosum, left and right crus).	One session per week for 4 weeks.
3	Renova ®	5000 shocks of 0.09mJ/mm^2^	300 shocks/min	900 shocks at left and right corpus cavernosum; 1600 shocks at left and right crus.	One session per week for 4 weeks.
4	Duolith® SD1 ultra (Storz Medical AG, Tägerwilen, Switzerland)	3000 shocks of 0.25mJ/mm^2^	6Hz	Distal penis (1000 shocks), base of penis (1000 shocks), and corporal bodies on perineum (500 shocks to each crura)	Twice weekly for 6 weeks
5	Duolith® SD1 ultra (Storz Medical AG, Tägerwilen, Switzerland)	3000 shocks of 0.15mJ/mm^2^	5Hz	Six treatment sites (distal, central and proximal part of each corpus cavernosum)	One session per week for 5 weeks.
6	Duolith® SD1 T-Top (Storz Medical, Tägerwilen, Switzerland)	1000 shocks of 20mJ/mm^2^, 15mJ/mm^2^ and 12mJ/mm^2^	5Hz	Shocks of 20mJ/mm^2^, 15mJ/mm^2^, 12mJ/mm^2^ were applied to the root of penis, to the shaft, and at a few millimeters proximal to the glans, respectively.	Twice sessions every other week for six weeks
7	LGT-2500B(Long Zhi-jie Ltd, Guangzhou, China)	1500 shocks of 1 bar	2Hz	300 shocks were delivered at each of the 5 treatment points (the distal, mid and proximal penile shaft, and the left and right crura).	Twice a week for 4 weeks

#### The quality of the included studies

The risks of bias in two RCTs were considered to be high owing to high dropout rate (25) and not performing blind method in performance (21). Three RCTs (10, 17, 28) had unclear risks of bias without details of randomization and/or blind method, and only one RCT was evaluated as being low risk of bias (18). According to MINORS (16), all non-comparative studies were 12 scores with lack of information of blind evaluation of endpoints and prospective calculation of the study size.

#### Evaluation of the effect of LI-ESWT on ED in terms of IIEF-EF and EHS, and its safety

**Figure 2:**
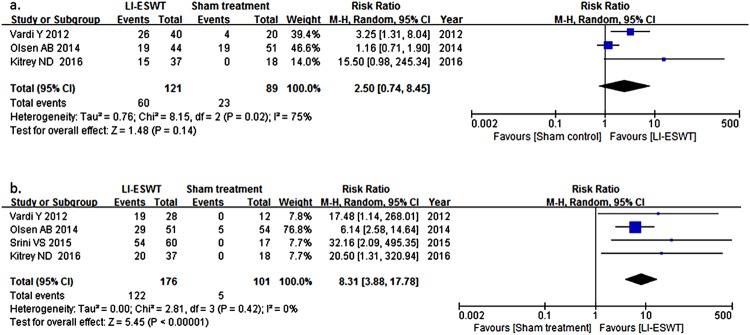
Forest plots of random effects model of risk ratio of effective treatment of LI-ESWT for ED in terms of International Index of Erectile Function-Erectile Function Domain (a) and Erectile Hardness Score (b).

Three RCTs (10, 17, 28) comparing IIEF-EF-based effective rate (ER) in patients receiving LI-ESWT (n= 121) and sham treatment (n=89) were included for the calculation of RR. Effective treatment was 2.50 [95°/o confidence interval (CI): 0.74-8.54] times higher in the LI-ESWT group than in the sham-controlled group at about 1 month after last session and heterogeneity may be substantial (p=0.02; I^2^: 75%). In terms of EHS, including 4 RCTs (10, 17, 25, 28) of 277 patients, RR is 8.31 with 95% CI ranging from 3.88 to 17.78 (p=0.42; I^2^: 0%), as seen in Figure-2. Funnel plot was asymmetrical showing publication bias. Sensitivity analysis in IIEF-EF revealed that the study by Olsen et al. (17) influenced heterogeneity significantly and when the study was excluded, I^2^ and p value was 24% and 0.25, respectively, while RR was 4.40 (95%CI: 1.18-16.38). Sensitivity analysis indicated that the result was stable in EHS.

**Figure 3:**
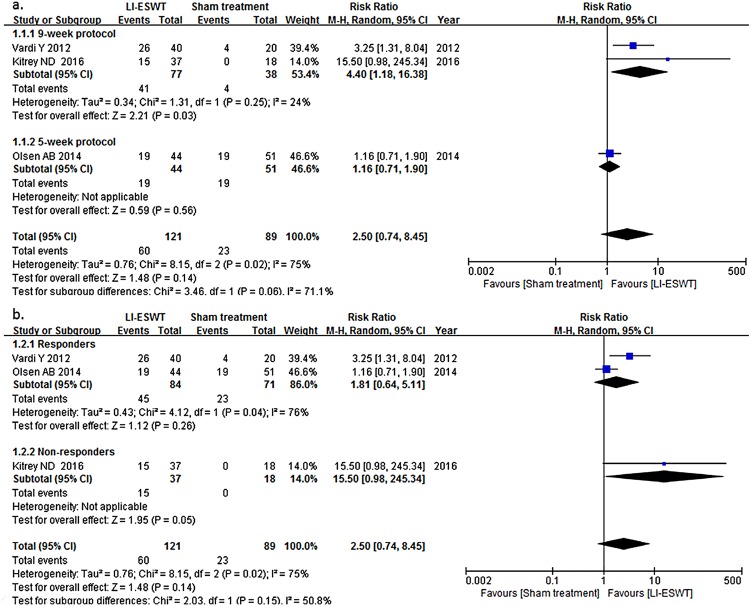
Relationship of clinical variables and treatment procedures in International Index of Erectile Function-Erectile Function Domain (IIEF-EF). (a) The studies using the 9-week protocol of LI-ESWT more possibly contributed to effective treatment (risk ratio [RR]: 4.40; 95% confidence interval [Cl]: 1.18-16.38; p=0.25), than using 5-week protocol (RR: 1.16; 95% Cl: 0.71-1.90), although it did not reach statistical significance (p=0.06). (b) LI-ESWT for PDE5I non-responders more possibly contributed to effective treatment (RR: 15.50; 95% Cl: 0.98-245.34), than for responders (RR: 1.81; 95% Cl: 0.64-5.11; p = 0.04), but it did not reach statistical significance neither (p=0.15).

**Figure 4:**
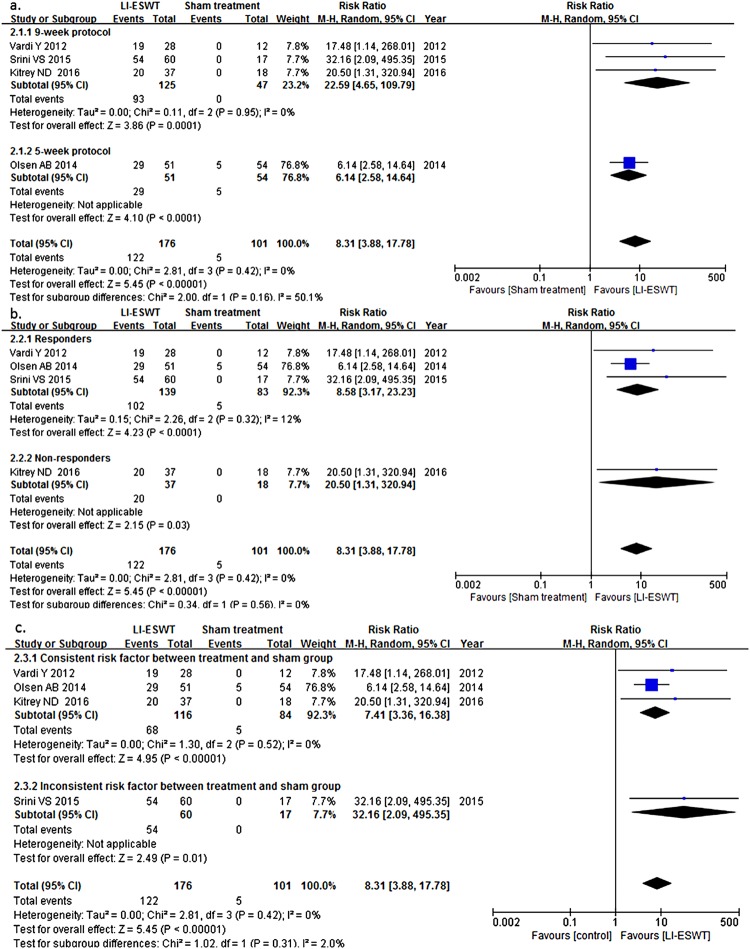
Relationship of clinical variables and treatment procedures in the Erection Hardness Score (EHS). (a) The studies using the 9-week protocol of LI-ESWT more possibly contributed to effective treatment (risk ratio [RR]: 22.59; 95% confidence interval [Cl]: 4.65-109.79; p=0.95), than using 5-week protocol (RR: 6.14; 95% Cl: 2.58-14.64), although it did not reach statistical significance (p=0.16). (b) LI-ESWT for PDE5I non-responders more possibly contributed to effective treatment (RR: 20.50; 95% CI:1.31-320.94), than for responders (RR: 8.58; 95% Cl: 3.17-23.23; p=0.32), but it did not reach statistical significance (p=0.56). (c) The outcome of studies with consistent risk factor of ED between treatment and control group is lower (RR: 7.41; 95% Cl: 3.36-16.38; p=0.52), than that with inconsistent risk factors (RR: 32.16; 95% Cl: 2.09-495.35), but it did not reach statistical significance (p=0.31).

In subgroup analysis, as seen in Figures 3 and 4, it was showed that in EHS 9-week protocol with energy density of 0.09mJ/mm^2^ and 1500 pluses (RR: 22.59; 95% CI: 4.65-109.79) was probably more effective than 5-week protocol with energy density of 0.15mJ/mm^2^ and 3000 pulses (RR: 6.14; 95% CI: 2.58-14.64) based on possibly substantial between-group heterogeneity, although the difference did not reach statistical significance. Similar result was observed in IIEF-EF with RR of 4.40 (95% CI: 1.18-16.38) in 9-week group and 1.16 (95% CI: 0.71-1.90) in 5-week group with the p value of subgroup differences being 0.06. In our analysis, LI-ESWT for PDE5i non-responders was more likely to contribute to effective treatment (RR: 15.50, 95% CI: 0.98-245.34, in IIEF-EF; RR: 20.50, 95% CI: 1.31-320.94, in EHS), than for responders (RR: 1.81, 95% CI: 0.64-5.11, in IIEF-EF; RR: 8.58, 95°/o CI: 3.17-23.23, in EHS), but difference was not statistically significant. To explain whether the consistency of risk factors of ED, such as age, cardiac disease, diabetes mellitus (DM), et al., between treatment and control group influenced analysis outcome, we summarized the baseline characteristics of study population from the 4 RCTs (Table-3) and performed subgroup analysis according to consistency of risk factors. It was found that the outcome of studies with consistent risk factor was lower (RR: 7.41; 95% CI: 3.36-16.38), than that with inconsistent risk factors (RR: 32.16; 95% CI: 2.09-495.35), but it did not reach statistical significance either (p=0.31).

**Table 3:** Baseline characteristics of study population in 4 RCTs for meta-analysis.

Study	Age[median(years), range]	EHS	IIEF	Diabetes	Hypertension	Heart disease	Smoking	Alcohol	Lipids
Vardi 2012 (10)	58 (27-72) vs. 57 (35-77)#	≤2	IIEF-EF < 19	30 vs. 30%#	ND	20 vs. 10%#	ND	NA	ND
Olsen 2014 (17)	59 (41 80) vs. 60(37–79)	<2	IIEF-EF < 20	18 vs. 13%#	33 vs. 37%#	4 vs. 11%#	ND	ND	NA
Srini 2015 (25)	NA	≤2	IIEF-EF < 18	ND	22.11 vs. 5% (p=0.0219)	3.16 vs. 25% (p=0.0003)	ND	23.16 vs. 47.5% (p=0.0074)	20 vs. 47.5% (p=0.0017)
Kitrey 2016 (28)	60 (28-78) vs. 64 (29-81)#	≤2	IIEF-EF ≤ 12	56.8 vs. 72.2%#	ND	48.6 vs. 38.9%#	ND	NA	ND

The former is a LI-ESWT group and the latter is a controlled group in the blank. **#**: No significant differences between groups. **ND:** no significant differences. **NA:** not applicable.

There was no reported severe complication, which needed medical intervention.

## DISCUSSION

Since 2010 when Vardi et al. (9) published the first literature on LI-ESWT for ED, most of published clinical studies (9-11, 17-28) on the topic have favored the modality with the ability of ameliorating patient's EF. It has been written in the EAU guideline as a potential first-line therapy for ED since 2013, although detailed recommendation has not been given since then. On the basis of current literatures, our metaanalysis also suggested that penile LI-ESWT probably represents an effective approach in the treatment of ED, when evaluated by using IIEF-EF and EHS. Hemodynamic improvement was measured objectively via flow mediated dilatation (FMD) technique in some well-designed RCTs (10, 28), although these data were limited and not suitable for cumulative analysis. In preclinical researches, it was revealed that LI-ESWT could induce angiogenesis, nerve regeneration, progenitor cell recruitment, endothelial functional improvement, tissue remodeling reverse, and microenviroment improvement to improve EF (29, 30). In addition, there was no adverse side effect reported. Overall, current evidences support LI-ESWT as a potential choice for ED clinically and preclinically.

Recently, two systematic reviews and metaanalyses (12, 13) on the topic were published and their conclusions were similar to ours. However, there were some limitations in their study designs. The study by Lu et al. (12) included seven RCTs on LI-ESWT for organic ED and ED associated with PD and CPP. Heterogeneity was evident pathologically and clinically among the three types of ED (31). Furthermore, a recent meta-analysis proved that PD-associated ED could not benefit from extracorporeal shockwave therapy (32). Therefore, the application possibility of cumulative results was reduced and subgroup analysis was not convincing enough when explaining the exact source of heterogeneity based on their inclusion criteria. It is more reasonable to separate different ED according to pathogenesis when the efficacy of LI-ESWT was evaluated. The other study by Angulo et al. (13) included single arm trials with evidence level 2, inevitably lowering the quality of their meta-analysis. Our meta-analysis focused on RCTs regarding organic ED with similar inclusion/exclusion criteria and excluded studies on CPP- and PD-associated disease. In our opinion, our design is the most reasonable among the three meta-analyses.

Different protocols of LI-ESWT likely influence its therapeutic effect on ED, and more frequent treatment and longer treatment course seems to be more effective, although there is no comparative trial to define the best protocol. Our analysis revealed that 9-week protocol with 12 sessions, which was proposed by Vardi et al. (9) and used most frequently, was more effective than 5-week protocol with 5 sessions. In a study evaluating additional shock wave therapy, it was demonstrated that “second round” LI-ESWT was essential for patients with poor response to the previous treatment (33). Nevertheless, shorter LI-ESWT protocols were also investigated because repeated visits to hospital and long duration of treatment could compromise patient's compliance (18, 25), and favorable results of short protocols were also proven (22-24). Therefore, additional comparative studies among different protocols are demanded to create an optimal protocol.

Energy density (2) and dose (30) of shock wave therapy are considered to be relative to physiological effect. As regard to penile tissue, high energy level causes apoptosis and collagenization of corporal smooth muscle with consequently deteriorating EF (34). When low energy shock wave therapy was conducted in the treatment of diabetic ED, EF was significantly improved with increased smooth muscle and endothelial content. Meanwhile, it was proven that improvement was more significant in the 300-shock group than in the 100- and 200-shock groups (30). The cumulative analysis revealed that 9-week protocol with energy density of 0.09mJ/mm^2^ and 1500 pulses (300 for each treatment site) seemed to be superior to 5-week protocol with energy density of 0.15mJ/mm^2^ and 3000 pulses (500 for each site). However, an optimal energy density and number of pulses could not be further defined separately because of limited literature. In fact, no agreement exists on optimal energy and dose range for LI-ESWT and current literature reveal that energy density and dose applied in the field of ED with promising effect usually range from 0.09mJ/mm^2^ to 0.25mJ/mm^2^ and 1500 to 5000 times (300-1600 shocks for each site) respectively. Direct comparative trials and more precise preclinical researches are essential to make optimal parameters.

Compared to second- and third-line therapy for ED, intracavernosal injection of vasoactive drugs and surgical implantation of penile prostheses, LI-ESWT is noninvasive and rehabilitative. Severe ED patients who didn't respond to the first-line therapy of PDE5is could benefit from the treatment (11, 19, 20, 28). Our analysis revealed that non-responders, moderate to severe EDs with baseline IIEF-EF ≤ 12, appeared to have more possibility to benefit from LI-ESWT than responders with baseline IIEF-EF <20. Interestingly, the only RCT (18) with low risk of bias suggested that although improvement in IIEF-EF in the LI-ESWT group didn't reach significant difference compared to the sham group as a whole, the former's elevated IIEF-EF score was significantly higher than the latter's in subgroup analysis of severe ED patients with Sexual Health Inventory for Men (SHIM) score 5-7. Nevertheless, underlying association between the severity of ED and therapeutic impact of LI-ESWT is not clear and requires further investigation.

In addition to baseline EF, other patient characteristics, such as age, DM, hypertension, heart disease, smoking and/or alcohol consumption, and lipid level, potentially influence the effect of LI-ESWT on ED and thus different proportion of age and comorbidities between the treatment and control group, among different RCTs, likely commits the result of a RCT and meta-analysis. Almost all included RCTs provided participant's information regarding age and comorbidities, but there was no further investigation to determine the impact of age and comorbidities on the effect of LI-ESWT. In the study by Srini et al. (25), comorbidities were inconsistent between the treatment and control group with higher incidence in the latter. Although RR appeared to be far higher in the Srini's study than the other three studies (10, 17, 28) having consistent comorbidities between the treatment and control group, our analysis still cannot answer the question on whether comorbidities are associated with the effectiveness of LI-ESWT because the difference did not reach statistical significance. Therefore, RCTs with stratification of age and comorbidities are needed to determine the impact of these factors on the effect of LI-ESWT for patients with ED.

Short-term effective treatment defined as “participant's score increased by at least 5 points more than baseline in IIEF-EF, or a patient (baseline EHS ≤2 pts) with EHS ≥3 pts at about 1 month after LI-ESWT” was accepted because most participants and RCTs could be included for meta-analysis. Data about the change of IIEF-EF after treatment in the manner of mean and standard deviation, which were used in the other two meta-analyses, were available in only one (18) of the five RCTs (35). The minimal clinically important difference (MCID) of IIEF-EF is considered to be ideal to assess the true clinical efficacy of an intervention (36) and has been gradually used in the clinical trials about LI-ESWT for ED (23, 28). With more RCTs being published, meta-analysis on the topic using MCID as evaluation criteria is essential in the future.

The initial and optimal functional time of LI-ESWT is still unclear. The effect of LI-ESWT appears to be time-dependent in clinical practice. In the studies using 9-week protocol, the majority of patients felt improvement in EF initially between the sixth and eighth session (10, 11). The efficacy reached peak at 4-6 weeks after all sessions, then declined (17, 20, 25). For about half of the patients, the positive effect would gradually wane over two years, most of whom were severe and diabetic ED patients (37). With aid of PDE5i, however, the peak effect could be showed at 6 months (23, 26). In the laboratory, it was identified previously that shockwave-induced neovascularization was evident at 4 weeks after the treatment and persisted for 12 weeks with angiogenesis-related factors beginning to rise in 1 week, keeping high for 8 weeks, and then decline at 12 weeks (3, 38). Based on aforementioned clinical and preclinical results, it is rational to evaluate the short-term effect on ED at about one month after LI-ESWT in the review.

There are few evidences on LI-ESWT for ED other than vasculogenic type. Nerve sparing prostatectomy ED could benefit from the method (27), while patients suffering from non-sparing nerve surgery probably not (20, 39). Nonetheless, it was revealed that LI-ESWT alone or combined with human adipose-derived stem cells (h-ADSCs) seemed to have ability to promote erectile function recovery in a rat model of ED with bilateral pelvic nerve injury (40, 41).

There are some limitations in this study. First, detailed individual patient data were not available from all the studies. In the review, not all included studies were used for meta-analysis due to heterogeneous endpoints. Nevertheless, the non-quantitative analyzed studies were listed as describable summary to support the use of LI-ESWT. Second, there was substantial variability among the studies, e.g., the severity of ED, age, comorbidity, device, energy density and distribution, frequency of treatment, interval between sessions, and aid of medication. These are confounding factors which likely influence efficacy and should be considered when defining optimal modality strategy. Third, although the confidence interval of RR exceeds the nullity line when efficacy was evaluated by IIEF-EF, the effective treatments were far more in the LI-ESWT group than in the controlled group in the Vardi's (10) and Kitrey's studies (28). After excluding the study by Olsen et al. (17) with obviously influencing stability of cumulative result, RR was 4.40 (95%CI: 1.18-16.38). The authors (17) explained that inconsistent results between using IIEF-EF and EHS were caused by a part of patients having some problems of understanding the questionnaires of IIEF-EF. Fourth, the quality and number of the eligible studies were relatively limited and none of the 4 RCT was elevated as low risk of bias. Meta-analysis on the basis of these RCTs likely influenced its reliability as level 1a evidence. Fifth, publication bias existed because of four factors: 1) only English and Chinese literatures were included; 2) other language, unpublished studies and conference abstracts were excluded; 3) inflated estimates by a flawed methodological design in smaller studies; 4) and/or a lack of publication of small trials with opposite outcomes. Sixth, objective measured results, and middle- and long-term effects were unavailable in our meta-analysis due to lack of sufficient data.

## CONCLUSIONS

In summary, LI-ESWT, as a noninvasive treatment, with potential short-term therapeutic effect on patients with organic ED irrespective of sensitivity to PDE5is. Owing to the limited number and quality of the studies, more large-scale, well-designed and long-term follow-up time studies are needed to confirm our analysis.
